# Oropharyngeal Microbiota in Frail Older Patients Unaffected by Time in Hospital

**DOI:** 10.3389/fcimb.2018.00042

**Published:** 2018-02-20

**Authors:** Victoria C. Ewan, William D. K. Reid, Mark Shirley, A. John Simpson, Steven P. Rushton, William G. Wade

**Affiliations:** ^1^South Tees Hospital, NHS Foundation Trust, Middlesbrough, United Kingdom; ^2^Institute of Cellular Medicine, Medical School, Newcastle University, Newcastle upon Tyne, United Kingdom; ^3^Marine Sciences, School of Natural and Environmental Sciences, Newcastle University, Newcastle upon Tyne, United Kingdom; ^4^Model, Evidence, Policy, School of Natural and Environmental Sciences, Newcastle University, Newcastle upon Tyne, United Kingdom; ^5^Centre for Immunobiology, Blizard Institute, Barts and The London School of Medicine and Dentistry, Queen Mary University of London, London, United Kingdom

**Keywords:** microbiota, aged, cross infection, comorbidity, oropharynx, frail elderly, respiratory tract infections

## Abstract

Respiratory tract infections are the commonest nosocomial infections, and occur predominantly in frailer, older patients with multiple comorbidities. The oropharyngeal microbiota is the major reservoir of infection. This study explored the relative contributions of time in hospital and patient demographics to the community structure of the oropharyngeal microbiota in older patients with lower limb fracture. We collected 167 throat swabs from 53 patients (mean age 83) over 14 days after hospitalization, and analyzed these using 16S rRNA gene sequencing. We calculated frailty/comorbidity indices, undertook dental examinations and collected data on respiratory tract infections. We analyzed microbial community composition using correspondence (CA) and canonical correspondence analysis. Ten patients were treated for respiratory tract infection. Microbial community structure was related to frailty, number of teeth and comorbidity on admission, with comorbidity exerting the largest effect. Time in hospital neither significantly changed alpha (*t* = −0.910, *p* = 0.365) nor beta diversity (CA1 *t* = 0.022, *p* = 0.982; CA2 *t* = −0.513, *p* = 0.609) of microbial communities in patient samples. Incidence of respiratory pathogens were not associated with time in hospital (*t* = −0.207, *p* = 0.837), nor with alpha diversity of the oral microbiota (*t* = −1.599, *p* = 0.113). Patient characteristics at admission, rather than time in hospital, influenced the community structure of the oral microbiota.

## Introduction

Over a quarter of all hospital-acquired infections in Europe affect the respiratory tract (European Centre for Disease Prevention and Control, 2013). Nosocomial respiratory tract infections increase the average cost of hospital care by up to 75% (Thompson et al., 2006), increase length of hospital stay by up to 12 days (Thompson et al., 2006; Redelmeier et al., 2010), require the prescription of broad-spectrum antibiotics which may act as a driver for antimicrobial resistance, increase mortality (Thompson et al., 2006; Burton et al., 2016), and are associated with loss of independence (Thompson et al., 2006). Approximately two thirds of these infections occur in patients who have not been intubated and mechanically ventilated in critical care (Health Protection Agency, 2012). The non-ventilated hospital population at risk is predominantly older, frail and characterized by multimorbidity (European Centre for Disease Prevention and Control, 2013; Burton et al., 2016).

The oropharynx is a major reservoir of infection for respiratory tract infections (Bassis et al., 2015), with the emergence of respiratory pathogens in the oropharynx being associated with subsequent pulmonary infection (Heo et al., 2008; Ewan et al., 2015; Yeh et al., 2016), via misdirection of these organisms into the respiratory tree (microaspiration) (Bassis et al., 2015; Dickson et al., 2017). Tracheal and oral colonization with respiratory organisms is associated with subsequent increased risk of ventilator associated and hospital acquired pneumonia, respectively (Ewan et al., 2015; Kalil et al., 2016). Respiratory tract infections such as hospital-acquired pneumonia arise, by definition, at least 48 h, and on average 11 days after hospital admission (Hanson et al., 1992). However, little is known about the dynamics of the oropharyngeal microbiota during this window of infectivity in the population of frail, older patients at risk of respiratory tract infection. Oropharyngeal samples from older pneumonia patients in a previous study (de Steenhuijsen Piters et al., 2016) showed increased relative abundances of *Streptococcus pneumoniae, Rothia*, and *Lactobacillus* species, but because samples were taken after the onset of pneumonia, it was unclear as to whether these changes represented cause or effect. By studying the oropharyngeal microbiota prior to the onset of infection, we investigated whether patients who subsequently develop RTI are identifiable, either by a reduction in diversity or dominance of particular organisms, as is seen in ventilated patients prior to the onset of infection (Fourrier et al., 1998; Yeh et al., 2016).

Reduction in alpha diversity (i.e., the number of species in a sample) and over-representation of gammaproteobacteria in particular are consistently seen across a variety of conditions, including exacerbations of chronic obstructive pulmonary disease (COPD) (Dickson et al., 2014; Wang et al., 2016), and in the gut microbiota in frail patients (Claesson et al., 2012), but it is unclear whether this reduction in diversity is key to the succession of respiratory pathogens. We investigated whether changes in diversity were observable in hospitalized patients prior to the onset of respiratory infection, in a group of patients with lower limb fractures (hip, femur, ankle). Operation is mandatory in patients with hip fracture, so the study patients were representative of a spectrum of frailty states and comorbidities. However, there was consistency in the course of each patient's medical episode (e.g., fall, fracture, operation, recovery). This allowed us to investigate how patient factors such as frailty, comorbidity and also dentition influenced the oropharyngeal microbiota.

We sought to understand (a) how the alpha- and beta- diversity of the oropharyngeal microbiota changed over time in hospital, (b) how the structure of the oropharyngeal microbiota was related to interactions between patient factors such as frailty, comorbidity and dentition, and (c) whether oropharyngeal bacterial diversity was associated with relative abundance of established respiratory pathogens over time.

## Methods

For further details see the online data supplement.

### Ethical statement

Ethical approval was granted by the Newcastle and North Tyneside 2 Research Ethics Committee (REC 08/H0907/84). The research was conducted as per the Mental Capacity Act 2005 (United Kingdom) guidelines, and written patient consent was obtained from patients, or their relatives if patients lacked mental capacity.

### Patient recruitment and sample collection

We took oropharyngeal samples in 2009–2011 from patients aged >65 years with lower limb fractures (hip, femur, ankle), as previously described (Ewan et al., 2015). Patients were recruited pre-operatively where possible, or on the first post-operative day. The day of admission was considered to be day 0. Exclusion criteria included immunosuppression within the last 3 months (immunosuppressive drugs, chemotherapy or radiotherapy or ≥10 mg prednisolone per day), acute illness, palliative care and community-acquired pneumonia (i.e., pneumonia arising within 48 h of hospital admission).

Subsequent oropharyngeal sampling was undertaken at days 3, 5, 7, and 14 after the first sample. Flocked swabs were used to sample the tongue and throat at days 1, 3, 5, 7, and 14 (or nearest working day) between 8.30 a.m. and 12 p.m., or 1–4 p.m. No special instructions were given to patients prior to sample collection. Throat swabs were taken from anterior faucial pillars, using a back-and-forth motion three times. Tongue swabs were taken by making three strokes posteriorly-anteriorly, then rotating the swab 180° and making a further three strokes. Swab tips were transferred into 2 ml microtubes, transported to the Health Protection Agency (HPA) within four h of collection, and stored at 2–8°C until DNA extraction within 48 h. Samples were anonymized and stored at −80°C after DNA extraction.

### Demographic data and identification of participants with RTI

Baseline demographic data were collected and the Charlson comorbidity score (Frenkel et al., 2014) was calculated. The number of teeth or teeth on dentures was recorded. We used three complementary scales to identify frailty: Rockwood's Clinical Frailty Scale (CFS) (Rockwood et al., 2005), the Barthel Index (which measures activities of daily living), and the Hierarchical Balance and Mobility scale (Rockwood et al., 2008) These were, to a degree, co-linear but focussed on different phenotypic manifestations of the syndrome. For example, the Barthel index only reduces from score of 20 once the person needs help with activities of daily living, while the clinical frailty scale increases as comorbidities increase, and as the person starts needing a nap in the daytime, despite being independent with activities of daily living. The direction and description of these scales is available in Supplementary Material (Supplementary Table 1).

Case notes and prescription charts were reviewed weekly during admission to identify new courses of antibiotics for RTI. RTI was recorded when antibiotics were started for pneumonia by the responsible clinician after 48 h in hospital (i.e., a clinical endpoint) either for hospital-acquired pneumonia or lower respiratory tract infection.

### Routine care

All patients (apart from two treated without operation) received peri-operative antibiotics (until August 2009 this comprised three doses of cefuroxime 750 mg 12 hourly; after August 2009 the regimen was changed to three doses of teicoplanin 400 mg 12 hourly). All patients received 4500 international units of tinzaparin subcutaneously, unless already anticoagulated on warfarin. Routine postoperative analgesia was co-codamol 30/500 mg four times daily. Patients were routinely screened for MRSA and decolonized with chlorhexidine mouthwash and antibacterial toothpaste if found positive. No specific oral hygiene policy was in operation at the time of the study and the study team did not undertake any oral hygiene intervention. Patients relied on nursing staff helping with oral hygiene and bringing equipment to their beds if unable to mobilize, and leaving equipment within their reach. Patients with dentures were given denture pots if these were available, but these did not necessarily contain fluid.

### Construction of the phylogenetic library

Automated extraction of total nucleic acids from clinical bacterial isolates was performed using a NucliSens® easyMAG™ platform (bioMerieux, France), according to the manufacturer's instructions. The extracted DNA samples were stored at −80°C. Amplification of the c500 bp V1-3 region of the 16S rRNA gene present in all bacteria was performed using fusion primers (5′-CCATCTCATCCCTGCGTGTCTCCGACTCAG-NNNNNNNNNNNN-AGAGTTTGATYMTGGCTCAG-3′) composed of three portions: a golay barcode (specific to a single sample), a template specific region [to the 16S rRNA gene, 27FYM (Frank et al., 2008) and 519R (Lane et al., 1985)] and Roche GS-FLX Titanium series adaptor portion A and B (which later binds to the DNA capture beads during sequencing) using Lib-L emPCR. We used 100 forward primers, each with a unique error-correcting golay barcode (Fierer et al., 2008) which could then be used to distinguish pooled samples after sequencing. The reverse primer was as follows: 5′-CCTATCCCCTGTGTGCCTTGGCAGTCTCAG-GWATTACCGCGGCKGCTG-3′). Amplification was performed by NewGene Ltd (Centre for Life, Newcastle upon Tyne), and was as follows: samples were quality checked, and DNA quantified using picoGreen and purified using AmPure. Amplicons were checked for size and purity using the Agilent DNA 1000 kit and the Agilent 2100 Bioanalyzer. Quant-iT Picogreen fluorescent nucleic acid stain (Invitrogen) was used to quantitate amplicons and then the amplicons were pooled into a library at equimolar concentrations (1 × 109 molecules/μl). emPCR and undirectional sequencing of the libraries was performed using the Lib-L kit and Roche 454 GS-FLX Titanium sequencer at the Centre for Genomic Research, Liverpool University.

### Sequence analysis

Sequence analysis was performed using the mothur suite v 1.34 and the Schloss standardized operating procedure (Schloss et al., 2011). Good quality sequences were aligned to the SILVA 16S rRNA reference alignment (Quast et al., 2013). Sequences were identified with reference to the Human Oral Microbiome Database v 13.0 (HOMD) (Chen et al., 2010) with a sequence identity threshold of ≥98.5%. Sequences were binned into operational taxonomic units (OTUs) at a sequence dissimilarity distance of 0.015 (average neighbor algorithm). Data were subsampled to 2130 reads. Sequence data generated in mothur were visualized using interactive Tree of Life (iTOL).

### Definitions of respiratory pathogens

We defined OTUs likely to contribute to RTI in this study as *Haemophilus influenzae, Streptococcus pneumoniae, Staphylococcus aureus, Enterobacteriaceae, Pseudomonas aeruginosa, Escherichia coli*, and *Klebsiella pneumoniae*.

### Statistical analyses

Analyses were undertaken in R (R Core Development Team, 2015) using the Vegan (CA, CCA) (Oksanen et al., 2018), nlme (LME) (Pinheiro et al., 2017), piecewiseSEM (structural equation modeling) and dplyr (data manipulation) packages.

We used either species richness or the Shannon index to measure alpha diversity (number of species within a sample). We examined beta diversity (the differences between composition of samples between patients through time since admission) using a combination of correspondence analysis (CA) and canonical correspondence analysis (CCA). CA is an ordination approach that seeks to reduce the dimensionality of the data in order to identify major trends in the variation in taxonomic composition across the data set. Patients with similar ordination scores have similar oropharyngeal communities, whilst those with different scores have very different communities. CCA is a constrained ordination technique which seeks to explain trends in beta diversity in relation to potential drivers (such as, in this study, age, comorbidity, frailty and presence of teeth). A permutation test (permutations = 999) analyzing the marginal effect was undertaken to assess the significance of the constraining variables. Prior to undertaking the CA and CCA, OTUs that only appeared in <5 samples were removed from the data set and then these data were subjected to a Hellinger Transformation.

We used piecewise Structural Equation Modeling to quantify the impact of frailty, comorbidities, age, dentition and hospitalization on the beta diversity of the OTUs present in the oropharyngeal microbiota, with patient as the random effect. We used linear mixed effect (LME) modeling to investigate relationships between microbiome characteristics, taxon richness, sample diversity, frailty, comorbidity and time since admission, with the patient as a random intercept.

## Results

### Baseline characteristics

Patient characteristics are described in Table 1. Of the 53 patients studied, 42 patients were community dwelling prior to admission, 7 were from residential or nursing care homes and 4 were transferred from another hospital setting after an in-hospital fall. Ten were treated with antibiotics for respiratory tract infection during the in-patient study period (and thus sometimes after the sampling period). All these patients had a clinical frailty score of five or more (and were thus considered “frail”). Of the other 43 study patients, 27 were frail and 16 were “fit.” Two patients (both fit) had ankle fractures, and the other 51 patients had hip/femoral fracture. One patient did not receive an operation (stable ankle fracture); of the others 16 had general anesthetic with endotracheal intubation and the other 36 had laryngeal mask airway or spinal anesthesia. Mean length of operation was 88 min. One patient screened positive for MRSA and was treated with standard decolonization therapy.

**Table 1:** Demographics of study cohort.

**Variable**	**Total cohort *n* = 53, Mean, (*sd*); Median (range); *n* (%)**	**RTI patients *n* = 10**	**No RTI *n* = 27 Frail**	**No RTI *n* = 16 Fit**
Age	82.9 (6.4)	83.5 (8.5)	82.8, (5.9)	83.2 (5.9)
Female	32 (59.3%)	5 (50%)	14 (52%)	13 (81%)
Clinical frailty score (1–9, high score = more frail)	5 (1–9)	5 (3–7)	5 (5–9)	3.5 (1–4)
Barthel index (0–20, high score = more independent)	20 (4–20)	19 (4–20)	19 (4–20)	20 (12–20)
HABAM score (0–67, mobility-high score = more mobile)	51 (18–65)	50 (18–61)	50 (29–55)	53 (20–65)
Charlson index (high score = more comorbidities)	5 (2–11)	6 (4–11)	5 (2–11)	5 (3–10)
Number of teeth	6 (0–28)	7 (0–27)	0 (0–26)	6.5 (0–28)
Denture wearing	38 (72%)	8 (80%)	21 (78%)	9 (56%)
Number of medications at admission	6 (0–19)	5.5 (0–11)	7 (0–19)	3.5 (0–12)
Proton pump inhibitor	15 (28%)	5 (29%)	6 (22%)	4 (25%)
Inhaled corticosteroids	4 (8%)	1 (6%)	2 (7%)	2 (13%)
Dementia	6 (11%)	2 (20%)	4 (15%)	0 (0%)
Community dwelling prior to fracture	42 (78%)	6 (60%)	20 (74%)	16 (100%)
Door to scalpel (hours)	21 (3–744)	22 (7–744)	20 (6–72)	24 (3–120)
Length of stay (days)	27 (5–265)	46 (13–140)	27 (5–265)	23.5 (11–65)
Died up to 90 days post discharge	14 (26%)	8 (80%)	6 (22%)	0 (0%)

*RTI, Respiratory tract infection; IMD, Index of multiple deprivation score. Median (range) presented if distribution skewed*. ^*^n = 52.

Even in the frailer groups, the median Barthel scores were high, suggesting that many patients could still manage their own activities of daily living (e.g., washing, dressing, toileting).

247 samples were available for analysis after sequencing. After normalization, 76/247 (31%) samples fell below the threshold (2130 reads) and were discarded from the analysis, leaving a total of 167 samples. Between three and five samples were available from 38 patients, while 11 patients had two samples and four patients only had one sample.

**Figure 1:**
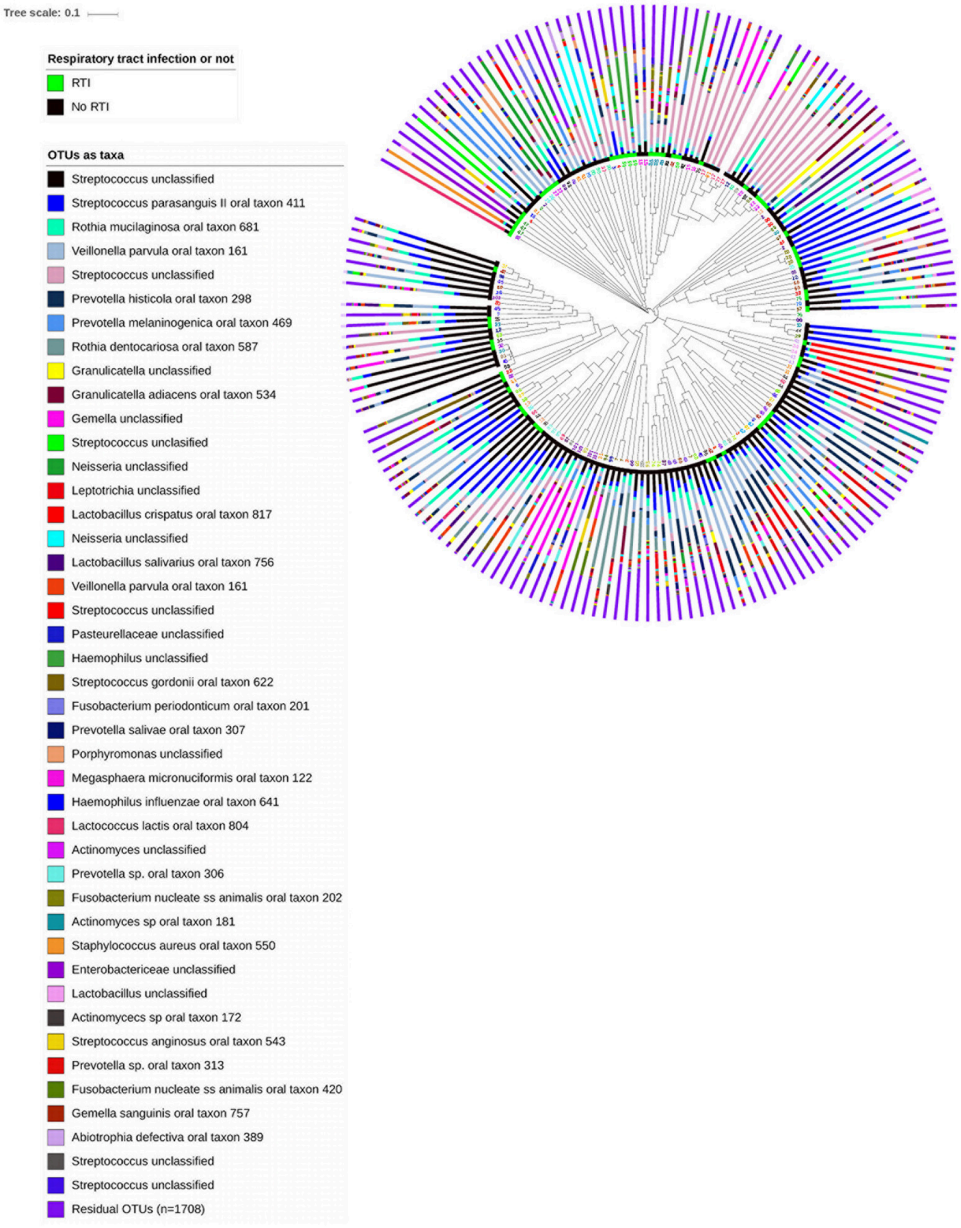
Unweighted hierarchical clustering analysis, using UPGMA algorithm (unweighted pair group method with arithmetic mean), of 167 oropharyngeal samples from 53 patients admitted to hospital with lower limb fracture. Adjacent to the branch ends, samples from patients who subsequently developed respiratory tract infection are highlighted in green, and those who did not were highlighted by the black bar. Stacked bar charts show the relative abundance of the 43 highest ranked OTUs (85% of the data) and the summed relative abundances of residual OTUs. Patient ID numbers are highlighted adjacent to dendrogram branch ends, and demonstrate similarity between samples from individuals.

From the 167 samples, 3,409 OTUs were identified, belonging to nine phyla, of which 1,741 were not singletons. The nine phyla were *Firmicutes* (48.3%), *Actinobacteria* (16.1%), *Proteobacteria* (13.5%), *Bacteroidetes* (12.9%), *Fusobacteria* (5.0%), *Spirochaetes* 0.7%, *Synergistetes* 0.3%, *Saccharibacteria* 0.2%, *Tenericutes* 0.1% and SR1 (0.1%). A further 2.6% of sequences were unclassified. Median Good's coverage was 0.997, with range 0.982–1.000, suggesting sufficient sequence depth. The oropharyngeal microbiota were highly individualized (Figure 1).

### Oropharyngeal microbiota structure over time in hospital

Species richness remained stable over time in hospital across all patients (*t* = −0.846252, *p* = 0.3992) irrespective of frailty scores. Similarly, the Shannon Index remained stable over time (*t* = −0.909811, *p* = 0.3649). We used the CA scores from patients with three or more samples to test whether beta diversity changed over time. There was no significant change in beta diversity in patients over time in hospital (CA1 *t* = 0.0221257, *p* = 0.982; CA2 *t* = −0.5125609, *p* = 0.609).

### Patient demographics and the oropharyngeal microbiota at admission

There were marked differences in OTU richness between patients (range of number of OTUs observed 11–208, mean 78, *SD* 42). Shannon index was inversely related to frailty at admission, (*t* = −3.057965, *p* = 0.0035), and positively associated with lower Charlson index (*t* = −2.694639, *p* = 0.0095), increased mobility score (*t* = 4.332602, *p* = 0.0001), and increased number of teeth (*t* = 2.882086, *p* = 0.0058).

**Figure 2:**
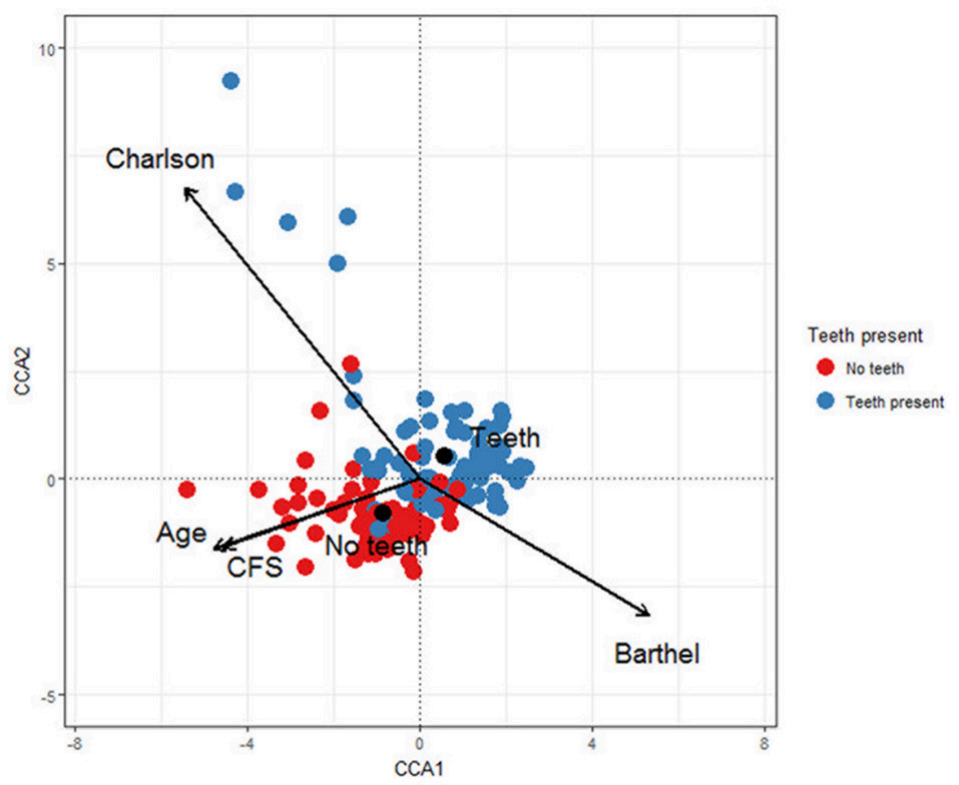
Canonical correspondence analysis showing the relationships between the microbial communities in hip fracture patients' mouths and Charlson score, Age, Clinical Frailty Score (CFS), Barthel score and whether teeth are present or absent.

In terms of beta diversity, the CCA plot (Figure 2) showed that Clinical Frailty Scale (χ^2^ = 0.069, *F* = 2.114, *p* < 0.001) and the presence or absence of teeth (χ^2^ = 0.104, *F* = 3.167, *p* < 0.001) were strongly associated with the main trend in taxon change along the first axis. The second trend in variation was related to Charlson index (χ^2^ = 0.109, *F* = 3.318, *p* < 0.001). Thus the oral microbiota of patients varied because of differences in frailty, comorbidity and presence of teeth. Extreme scores were observed for patients along the second axis, suggesting that the microbiota exhibited most differences at higher levels of comorbidity. Some samples from this area originated from two persons treated for RTI during the sampling period. The OTUs associated with negative CA2 scores at the extreme end of the tail were *Lactobacillus oris* (OTU 180), *Lactobacillus crispatus* (OTU 15) and the respiratory pathogen *S. aureus* (OTU 33) while the OTUs associated with positive CA scores were *H. influenzae* (OTU 27) and *Enterobacteriaceae* spp. (OTU 34) (Supplementary Figure 1).

**Figure 3:**
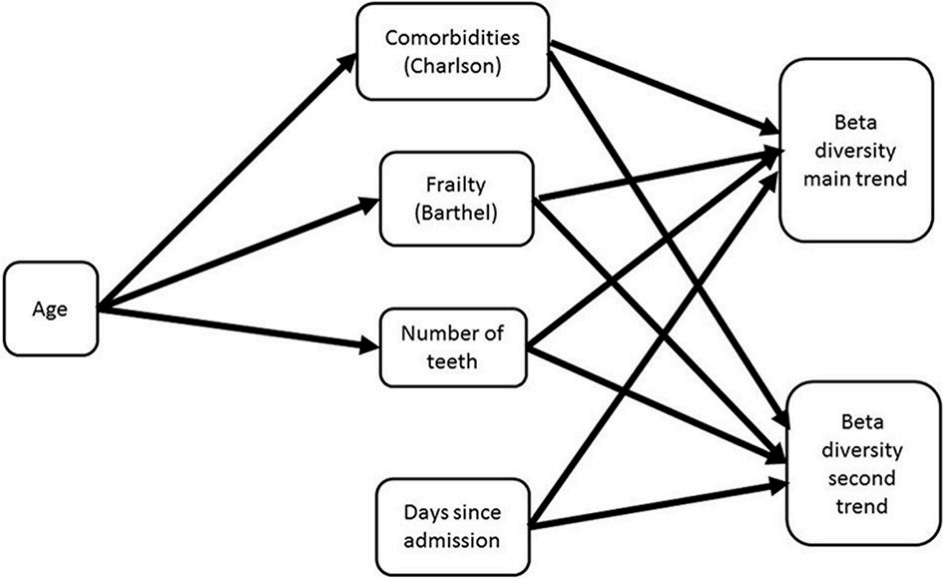
Hypothetical path diagram for a structural equation model analyzing the impacts of age, patient health status, and duration of time in hospital on the beta diversity of the oral microbiome as measured in terms of a correspondence analysis of OTUs collected from patients from admission to discharge or death (whichever occurred first).

**Figure 4:**
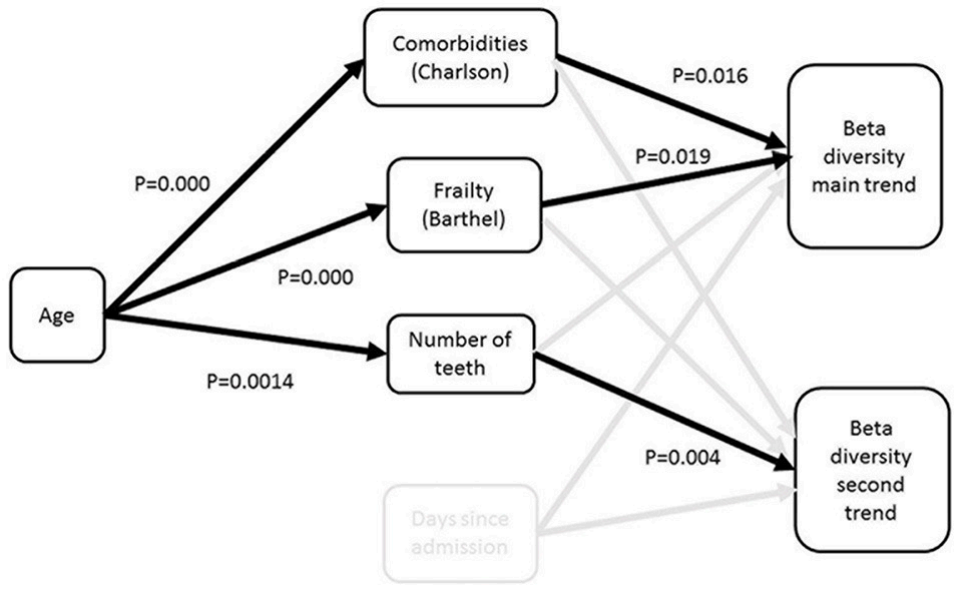
Path diagram for best fit structural equation model. The model demonstrates that the major trend in beta diversity is related to the extent of frailty and comorbidities, which are both related to age; the second trend is related to the number of teeth. There was no impact of time since admission on the trends in beta diversity.

The direct and indirect contributions of patient age, frailty, comorbidity and time in hospital to beta diversity were further analyzed using piecewise structural equation modeling (SEM). The hypothetical model is shown in Figure 3. The main trend in beta diversity represented by the first axis of the correspondence analysis of OTUs was related to the Charlson (*z* = −2.5105, *p* = 0.0157) and Barthel scores (*z* = 2.2261, *p* = 0.0311). In common with the univariate and multivariate analyses described above, this axis was not related to the duration in hospital (*z* = 1.1543, *p* = 0.2504), number of teeth (*z* = 0.2131, *p* = 0.8322) or clinical frailty scale (*z* = 0.3200, *p* = 0.7503). Axis two on the other hand was related to the Charlson index (*z* = −2.0192, *p* = 0.0494), and to number of teeth (*z* = −2.8192, *p* = 0.0071), but not to duration of time since admission (*z* = −0.1497, *p* = 0.8812), or Barthel score (*z* = 1.1548, *p* = 0.2542) or CFS (*z* = 0.966, *p* = 0.3392). Barthel, clinical frailty scale and Charlson scores were related to age (*z* = 19.428, *p* = 0.000; 11.586, *p* = 0.000 and *z* = 7.6443, *p* = 0.0000 respectively). The path diagrams showing observed significant pathways to effect is shown in Figure 4. Age clearly contributed to the trends in beta diversity through its effects on comorbidity and Charlson score, as older patients tended to be frailer and have more co-morbidities.

### Oropharyngeal bacterial diversity and respiratory pathogens over time in hospital

The OTUs likely to contribute to RTI in this study were OTUs 27 (*H. influenzae*), OTU 33 (*S. aureus*), and OTUs 34, 55, 72, and 129 (Enterobacteriaceae, unclassified). Other OTUs known to act as respiratory pathogens (e.g., *P. aeruginosa, E. coli*, and *K. pneumoniae*, see Supplementary Table 2) were present very rarely and in very low relative abundances (<0.001%) in this cohort, and were therefore not included in the analysis. We were unable to reliably identify *S. pneumoniae* and thus this organism is not included in analysis.

Respiratory pathogens were present in 38/167 samples (23%). The distribution of relative abundances of respiratory pathogens was highly positively skewed (median 0.48, range 0.04–89.1), with only 11/167 (7%) containing >5% respiratory pathogens. There were no obvious changes in the microbiome prior to first appearance of respiratory pathogens. The mixed effect models showed that there was no increase in relative abundances of respiratory pathogens over time in individual patients whilst in hospital (*t* = −0.206605, *p* = 0.8367). While RTI was associated with higher relative abundances of respiratory pathogens (*t* = 3.003900, *p* = 0.0041), there was no association between RTI and oropharyngeal Shannon index (*t* = −1.636156, *p* = 0.108). Neither were higher relative abundances of respiratory pathogens significantly associated with sample diversity (*t* = −1.598932, *p* = 0.1126). Thus samples with lower diversity were not more likely to contain respiratory pathogens.

## Discussion

This is the most comprehensive serial analysis to date of oropharyngeal samples taken from older hospitalized patients across a spectrum of frailty. We have shown that in this relatively well cohort of non-ventilated patients, oropharyngeal alpha and beta diversity remained relatively stable over the early admission period and that the main drivers of beta diversity at admission were frailty, comorbidity and number of teeth. While there are no directly comparable studies, two longitudinal studies of sputum samples from COPD patients demonstrated similar stability of the microbiome over time (Huang et al., 2014; Wang et al., 2016). Overrepresentation of respiratory pathogens did not appear to occur solely as a result of time in hospital. These data challenge the idea that time is the dominant factor shaping the oropharyngeal microbiota in this population.

Loss of species richness was associated with increased frailty, similar to other studies of gut microbiota (Claesson et al., 2012; Jackson et al., 2016). However because a proportion of non-RTI participants experienced similar frailty to our RTI participants, we were able to demonstrate that loss of species richness in itself was not associated with the occurrence of respiratory pathogens, nor with subsequent respiratory tract infection. This is in contrast to a previous study which found reduced oropharyngeal diversity in older pneumonia patients, but whose controls were healthy community dwelling older people (de Steenhuijsen Piters et al., 2016). Lower diversity communities are generally thought to be less resilient to invasion by infecting pathogens (Keesing et al., 2010), and these results might suggest that the organisms concerned are occurring due to overgrowth or stagnation (loss of flow) rather than invasion, in keeping with a previous study (Palmer et al., 2001). However we did not examine total bacterial load, and therefore the colonization resistance issue, pertinent to *Clostridium difficile* infection in the gut (Vincent et al., 2013), and which may be important here, was not addressed.

Our findings support the idea that, in frailer patients, “hospital” pathogens may be present at admission, which may have implications for the time dependent definition of nosocomial respiratory infection occurring after 48 h (American Thoracic Society and Infectious Diseases Society of America, 2005).

There are limitations to these data. While the study is unique, the number of patients studied was small. Older patients are particularly vulnerable after admission to hospital and recruitment is difficult, cognitive impairment notwithstanding (which may be in part why this population is understudied). These patients were too frail for bronchoscopy, therefore the diagnosis of respiratory tract infection was based on clinician-initiated antibiotics, and there were few sputum samples available with which we could compare oropharyngeal samples. However, there was a significant association between respiratory tract infection and prior detection of respiratory pathogens in the oropharyngeal flora (despite the lack of data on pneumococcus), corroborating this method of identifying cases of respiratory tract infection to some degree. In addition, oropharyngeal samples have been found to be comparable to sputum samples in exacerbation of COPD (Liu et al., 2017). There are also semantic difficulties because technically, though we would not consider these patients to have developed ventilator associated pneumonia, some were intubated and mechanically ventilated, albeit for a short period, intraoperatively. Nevertheless this cohort represents a different demographic group to the ventilated critical care patients, being older, frailer and less acutely unwell. However these issues do highlight some of the difficulties with labeling “types” of pneumonia.

Future research ought to include qPCR as a measure of total bacterial load to investigate whether respiratory pathogens occur as a result of loss of colonization resistance, or whether decreased mechanical removal of oral contents via swallowing impairment plays a greater role. We did not systematically assess swallowing as a covariate, one of the strongest risk factors for hospital-acquired respiratory tract infections (Cabré et al., 2014). However, anecdotal observations made during the study suggested an important role for dysphagia in the occurrence of respiratory pathogens (data not shown). Systematic identification of patients with swallowing problems combined with immunological data are likely to be key to understanding why dysbiosis of the oropharyngeal microbiota occurs. Credible (non-RCT) evidence suggests that four times daily oral hygiene may reduce the rates of nosocomial RTI (Quinn et al., 2014), and such programmes are being instigated across a number of hospitals in the United States. This is an another important avenue for research in this area.

## Conclusions

Host factors, rather than time in hospital, determined the structure of the oropharyngeal microbiota in non-ventilated older patients with lower limb fracture. The reduction in alpha diversity seen in frailer patients was neither significantly associated with the occurrence of respiratory pathogens nor with RTI.

## Author contributions

VE, WW: Conceived and designed study; VE: Performed the study; WW, VE: Performed the microbiome analysis; WR, SR, MS, VE: Performed statistical analysis; VE, WR, SR, WW, AS: Wrote the paper; AS: Advised on nosocomial infection; All authors: Critical review of paper.

### Conflict of interest statement

The authors declare that the research was conducted in the absence of any commercial or financial relationships that could be construed as a potential conflict of interest.

## Supplementary material

Supplementary Figure 1 Correspondence analysis showing “site” and “OTU” scores.

Supplementary Table 1 Descriptions of clinical covariates used in the study.

Supplementary Table 2 Presumed causative organisms detected from 835 culture-positive patients with hospital-acquired pneumonia from the US ATLAS database.
