# Supplementary figures and images for: Oropharyngeal Microbiota in Frail Older Patients Unaffected by Time in Hospital
Source: Front Cell Infect Microbiol. 2018 Feb 20;8:42. doi: 10.3389/fcimb.2018.00042 (PMC5826060; doi:10.3389/fcimb.2018.00042)

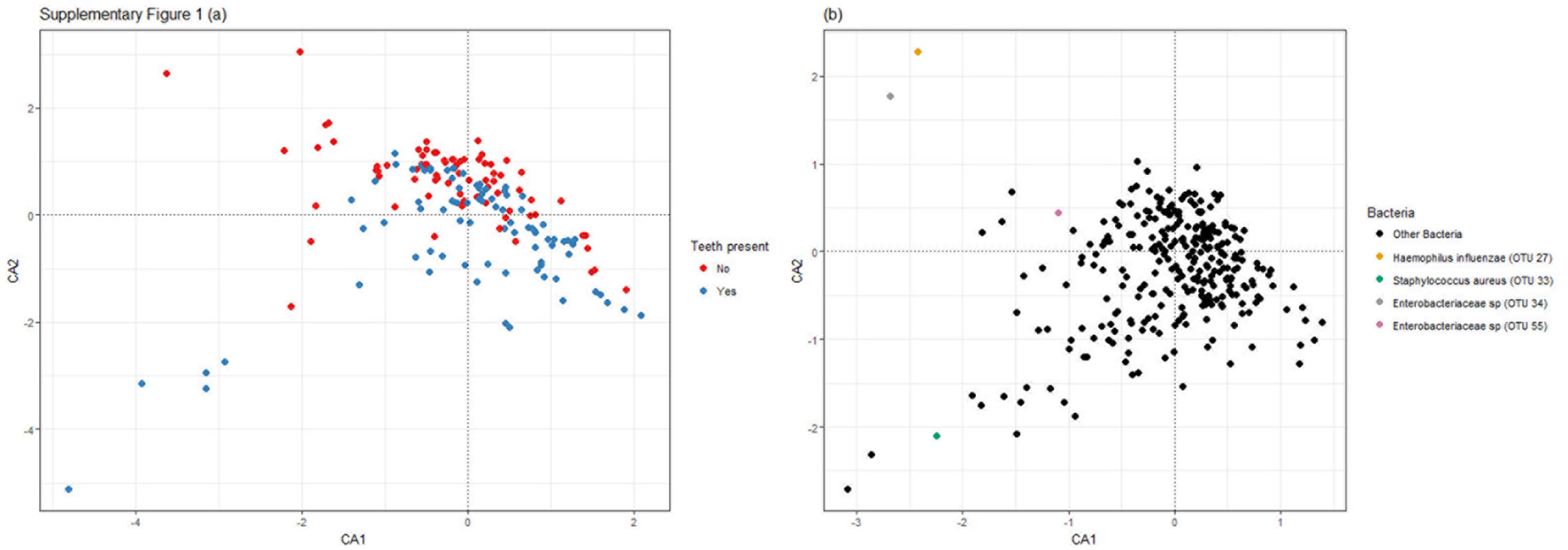

Supplement: Supplementary Figure 1 — Correspondence analysis showing “site” and “OTU” scores. [file Image1.TIF]
