# Supplementary material for: Oropharyngeal Microbiota in Frail Older Patients Unaffected by Time in Hospital
Source: Front Cell Infect Microbiol. 2018 Feb 20;8:42. doi: 10.3389/fcimb.2018.00042 (PMC5826060; doi:10.3389/fcimb.2018.00042)
Supplement: Supplementary Table 1 — Descriptions of clinical covariates used in the study. [file DataSheet1.DOCX]

Supplementary Material

Oropharyngeal microbiota in frail older patients unaffected by time in hospital

Victoria C Ewan, William Reid, Mark Shirley, A. John Simpson, Steven Rushton, William G Wade

*** Correspondence:** V. Ewan: Victoria.ewan@ncl.ac.uk

# Supplementary Tables

**Supplementary Table 1. Descriptions of clinical covariates used in the study**

| Covariate | Description of Scale | Advantages and Limitations |
| --- | --- | --- |
| Clinical frailty scale | 1= fittest to 9 = moribund; A score of 4 indicates that the person requires a nap during the day but needs no help with housework or shopping etc, while a score of 5 would indicate that help was required for heavier activities of daily living. | Distinguishes between fitter patients. However the lack of linearity of the CFS score means that large clinical changes are observed especially around scores 4-6 with little numeric change in the score. |
| Charlson comorbidity score | 0=fewest comorbidities, increases with certain illnesses (e.g. cancer, HIV) and age. | Only certain illnesses contribute to the score, however it has been validated across many populations. Predicts risk of death in next 10 years. |
| Hierarchical Assessment of Balance and mobility (HABAM) | Highest score of 67, declines with poorer mobility. | Sensitive to small differences in mobility status. |
| Barthel index | Commonly used in routine clinical practice. Score=20 if the patient can independently perform activities of daily living (e.g. toileting, grooming, feeding), while lower scores mean a loss of independence. | Emphasises variation in dependent persons. Does not distinguish housebound but independent from much fitter persons. |

**Supplementary Table 2. Presumed causative organisms detected from 835 culture-positive patients with hospital-acquired pneumonia from the US ATLAS database**

| Organism detected | % patients |
| --- | --- |
| *S. aureus* | 47.1 |
| *S. pneumoniae* | 3.1 |
| Streptococcus non group | 13.9 |
| Other Gram positive | 8.1 |
| *Haemophilus* sp. | 5.6 |
| *Pseudomonas* sp. | 18.4 |
| *Klebsiella* sp. | 7.1 |
| *Escherichia* sp. | 4.7 |
| *Enterobacter* sp. | 4.3 |
| *Acinetobacter* sp. | 2.1 |
| Other Gram negative | 3.7 |

**
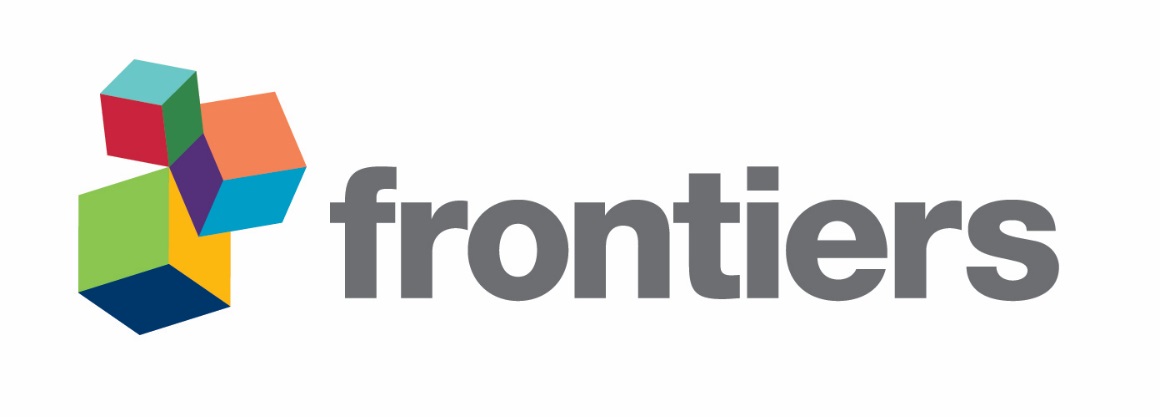
**
